# Opposite, bidirectional shifts in excitation and inhibition in specific types of dorsal horn interneurons are associated with spasticity and pain post-SCI

**DOI:** 10.1038/s41598-017-06049-7

**Published:** 2017-07-19

**Authors:** Olga Kopach, Volodymyr Medvediev, Volodymyr Krotov, Anya Borisyuk, Vitaliy Tsymbaliuk, Nana Voitenko

**Affiliations:** 1 Bogomoletz Institute of Physiology, Kyiv, Ukraine; 2 Institute of Neurology, University College London, London, UK; 3 Romodanov Institute of Neurosurgery, Kyiv, Ukraine

## Abstract

Spasticity, a common complication after spinal cord injury (SCI), is frequently accompanied by chronic pain. The physiological origin of this pain (critical to its treatment) remains unknown, although spastic motor dysfunction has been related to the hyperexcitability of motoneurons and to changes in spinal sensory processing. Here we show that the pain mechanism involves changes in sensory circuits of the dorsal horn (DH) where nociceptive inputs integrate for pain processing. Spasticity is associated with the DH hyperexcitability resulting from an increase in excitation and disinhibition occurring in two respective types of sensory interneurons. In the tonic-firing inhibitory lamina II interneurons, glutamatergic drive was reduced while glycinergic inhibition was potentiated. In contrast, excitatory drive was boosted to the adapting-firing excitatory lamina II interneurons while GABAergic and glycinergic inhibition were reduced. Thus, increased activity of excitatory DH interneurons coupled with the reduced excitability of inhibitory DH interneurons post-SCI could provide a neurophysiological mechanism of central sensitization and chronic pain associated with spasticity.

## Introduction

Spasticity is one of the most severe complications after spinal cord injury (SCI) or trauma, representing chronic motor deficit attributed with muscle spasms, hyperreflexia and impaired locomotion, which develops in up to 80% of the SCI patients^1–4^. With the irreversible motor dysfunctions, spasticity remains with limited options for an effective treatment focused primarily on reducing negative influences on an individual’s quality of life^5^. However, chronic pain often progressively develops within months after SCI and could expand to severe and unceasing pain syndrome either rapidly or through various patterns of delayed appearance^5, 6^. This pain is poorly treatable, which is, at least partly, due to poorly understood mechanisms of pain chronification.

It has emerged that the central mechanism of spasticity – hyperexcitability of motoneurons below lesion mediated by the plateau potentials and self-sustained firing – is causally related to changes in sensory processing in the spinal cord^7, 8^. The primary afferent inputs and activity of the wide dynamic range interneurons in response to either noxious or innocuous stimuli were both augmented post-SCI^9–11^, arguing for the hyperexcitability of the dorsal horn (DH), the area for sensory input integration and processing. Pathological signalling in sensory interneurons could involve a variety of impairments related to SCI, such as an upregulation of sodium channels^12, 13^, an increase in the extracellular glutamate level^14^, a reduced GAD expression^15^ and a shift in chloride gradient^16^. Notwithstanding the reported impairments at the cellular level, a neurophysiological explanation of pain remains elusive. Here we test the hypothesis that the pain mechanism involves changes in the DH circuitry with the key question how the overall hyperexcitability post-SCI relates to a high heterogeneity of DH interneurons.

To address this we combined electrophysiology with behavioural testing in experimental modelling of SCI in rats. Our findings proposed two complementary mechanisms contributing to the hyperexcitability of the DH post-SCI – increased excitability of excitatory lamina II interneurons and suppressed activity of inhibitory lamina II interneurons – that provide novel insights into neurophysiological mechanisms underlying chronic pain.

## Results

### Spasticity and pain post-SCI

**Figure 1:**
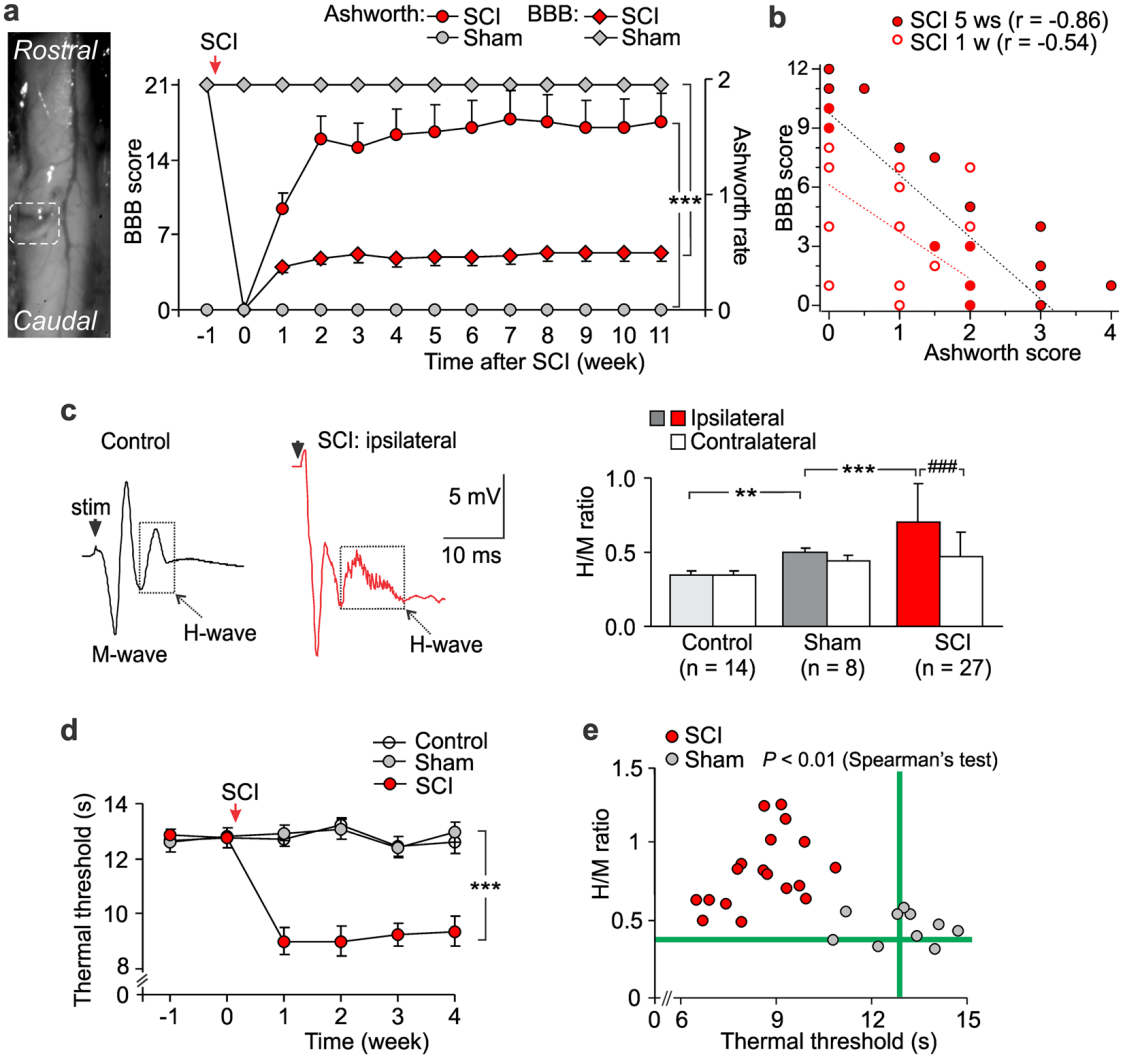
The SCI-induced spasticity correlates with chronic pain. (**a**) Left, image of the spinal cord immediately after the lesion (white box indicates the lesion site, Th_11_). Right, summary of the BBB (left Y axis) scaling of animal locomotion and the Ashworth rate (right Y axis) of long-lasting impairments in muscle tone on the ipsilateral side postoperatively (n = 32 SCI-injured rats, n = 14 sham-operated animals). ***P < 0.001 (two-way repeated analysis of variance and Bonferroni post-hoc test). (**b**) The BBB score estimated for each animal at week 1 and week 5 post-SCI (n = 32 animals) plotted against its Ashworth scoring value. The Pearson correlation coefficients (r) are indicated. (**c**) Left, examples of the H-reflex recordings, demonstrating the M- and the H-wave responses in control and post-SCI at ~1 month postoperatively. Right, the ratio of the H- to the-M-wave amplitudes in different experimental groups. ^###^ *P* < 0.001 (paired *t*-test), **p < 0.01, ***p < 0.001 (unpaired *t*-test). (**d**) The time course of changes in the thermal nociceptive threshold of the ipsilateral hindpaw in different groups of animals, demonstrating experiencing of the long-lasting pain post-SCI (n = 11 control, n = 14 sham-operated rats and n = 15 animals post-SCI). ***P < 0.001 (ANOVA with Bonferroni post-hoc test). (**e**) The H-reflex measurements (the H/M ratio) plotted against the thermal nociceptive threshold estimated for a respective animal at the time-point of ~1 month postoperatively for the sham-operated (n = 10 animals) and the SCI groups (n = 18 animals). The Spearman’s test significance is indicated. Green lines indicate the average parameters in control animals (no surgery). Data are expressed as mean; error bars, SEM.

The SCI-injured rats were examined for the motor deficit of hindlimb and chronic pain postoperatively. Each animal displayed severe motor dysfunction of the hindlimb ipsilateral to hemisection side (n = 32), including impaired joint movements with increased muscle tone (Table 1, see Methods Section), impaired coordination between corpus and the hindlimb, resulting in paralytic posture. The deficit developed rapidly (within 1 week post-SCI) and remained steadily for at least 2–3 months by exceeding on average the level 1 on the Ashworth rating scale and lowering the level 7 on the BBB score (*p* < 0.001; Fig. 1a). The regression analysis demonstrated a strong correlation between the BBB and the Ashworth scores that gradually increased with a time post-SCI: the Pearson correlation coefficient was –0.54 (*p* < 0.01) at week 1 and –0.86 at week 5 post-SCI (*p* < 0.001, n = 32; Fig. 1b). Consistent to the increased muscle tone observed in the SCI-injured rats, the H-reflex recordings (Fig. 1c) demonstrated the impaired sensorimotor integration post-SCI. The ratio of the H- to the M-waves was markedly increased on the ipsilateral side in the SCI-injured rats at ~1 month post-SCI (by ~33% compared with contralateral side, *p* < 0.001, paired *t*-test, n = 27 rats and by ~29% compared with average ratio on the ipsilateral side of the sham-operated group, n = 8 animals, *p* < 0.001, unpaired *t-*test; Fig. 1c).

**Table 1:** Scoring for the BBB and the Ashworth rating scales in unilateral SCI in rats.

Score	Criteria	
	BBB	Ashworth
0	no hindlimb movement	no increase in muscle tone
1	slight (less than half movement) of one or two joints (the hip and/or the knee)	slight increase in tone when hindlimb is moved in flexion or extension
2	extensive movement of one joint (more than half of the normal range of joint motion)	more marked increase in tone, with limb still easily flexed
3	extensive movement of two joints	considerable strength of tone with difficult passive movement
4	slight movement of all three joints	hindlimb rigid in flexion or extension
5	slight movement of two joints and extensive movement of the third	
6	extensive movement of two joints and slight movement of the third	
7 and above	extensive or full movement of all joints, including additional levels of plantar foot placement and/or weight support

Experimental SCI was reported to increase peripheral sensitivity to various stimulus modalities^17–19^ that may give rise to chronic pain. Therefore, we next examined changes in peripheral sensitivity of each hindlimb in the SCI-injured rats. We observed peripheral nociceptive hypersensitivity to thermal stimulation on the ipsilateral (Fig. 1d) and contralateral sides (Fig. S1) that developed shortly (within 1 week) and persisted over time post-SCI (the thermal threshold decreased by ~30%, n = 15 SCI-injured rats, *p* < 0.001 compared with the sham-operated group, unpaired *t-*test, n = 14). Moreover, the thermal pain hypersensitivity correlated with changes in the H-reflex (the Spearman correlation coefficient was –0.48, *p* < 0.01 n = 10 rats of the sham-operated group and n = 18 animals post-SCI; Fig. 1e). Chronic pain post-SCI could develop into unceasing, debilitating pain becoming intolerable, as suggested here by a self-mutilatory behaviour (autotomy), which some animals (about 15%) displayed later (2–3 months) postoperatively (Fig. S1B). Autotomy was directed ipsilaterally and typically proceeded with morphological changes, including skin reddening, tissue swelling, and tumour; it was associated with severe muscle spasm state and has been suggested to reflect intolerable chronic pain in animal studies^17, 20^. Neither sham-operated nor control animals exhibited mutilation (Fig. S1B). However, we failed to evidence peripheral mechanical hypersensitivity and allodynia post-SCI when measured the mechanical threshold with von Frey monofilaments; peripheral responses to plantar mechanical stimulation in the SCI-injured animals were comparable to those of the sham-operated group regardless innocuous or noxious stimuli were applied (Fig. S2).

### The DH hyperexcitability post-SCI

**Figure 2:**
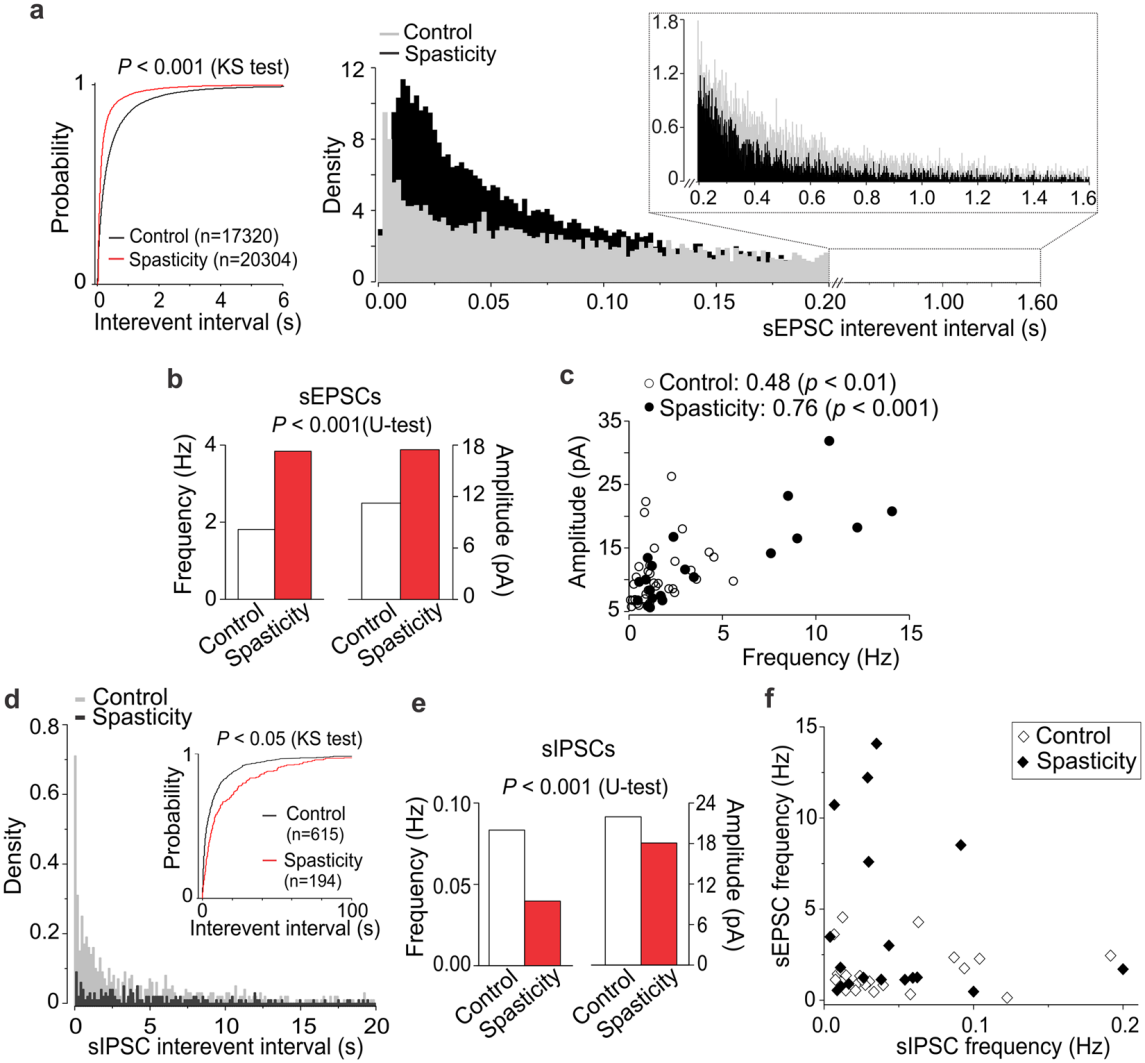
The SCI-induced hyperexcitability of the DH results from boosted excitation and synaptic disinhibition. (**a**) Voltage-clamp recordings in the DH interneurons (Vh = −70 mV) unveiled boosted circuitry excitation post-SCI. Cumulative probability plots (left) and the probability density functions for the inter-event interval of sEPSCs (right) demonstrate the high-frequency sEPSCs post-SCI and opposite the low-frequency sEPSCs in control (insert). (**b**) Summary for the median frequency (left Y axis) and the median amplitude (right Y axis) of sEPSCs in control and spasticity. (**c**) The median sEPSC frequency plotted against its median amplitude, estimated for each individual neuron (n = 34 cells from control and n = 21 cells from spastic animals). The Spearman correlation coefficients and the Spearman’s non-parametric test significance are indicated. (**d**) Voltage-clamp recordings of sIPSCs in the DH interneurons (Vh = 0 mV) revealed the decreased density of inhibitory currents post-SCI. Cumulative probability plots for the sIPSC inter-event interval in experimental groups (insert). (**e**) Summary for the median frequency (left Y axis) and the median amplitude (right Y axis) of sIPSCs in different conditions. (**f**) The median frequency of sEPSCs plotted against the median frequency of sIPSCs recorded from the same neurons in control (n = 24 cells) and spasticity (n = 18 cells). The Kolmogorov-Smirnov (KS) or the Mann-Whitney U-test significance is indicated.

The DH lamina I–II is the primary area for nociceptive input integration and pain processing. The hyperexcitability of motoneurons after SCI has been associated with changes in sensory transmission^8^. To test if spasticity and pain post-SCI are associated with the hyperexcitability of the DH, the voltage-clamp recordings were made in lamina II interneurons from adult rats experiencing long-lasting (1–2-month) spasticity (only animals with the level 3 to 4 on the Ashworth score were taken, with a clear spasm state). In total, 17320 sEPSCs were recorded in 36 interneurons from control (n = 7 rats), whereas 20304 events – in 22 ipsilateral interneurons from spastic animals (n = 6 rats). The median inter-event interval of sEPSCs was 205 ms in control, but 78 ms in spasticity (*p* < 0.001, the KS- and U-tests; Fig. 2a), indicating the increased frequency post-SCI (from 1.8 Hz to 3.8 Hz, increase by ~116%; Fig. 2b). There were predominant high-frequency sEPSCs in spasticity while the low-frequency sEPSCs in control (Fig. 2a, right). The median amplitude of sEPSCs was increased (from 11.2 pA in control to 17.5 pA in spasticity, increase by ~56%, *p* < 0.001, the KS- and U-tests; Fig. 2b). Changes in both frequency and amplitude were correlated (*p* < 0.05, the Fisher r-to-z transformation compared to control): the Spearman’s rank correlation coefficient was 0.76 in spasticity (n = 21, *p* < 0.001) *vs*. 0.48 in control (n = 34, *p* < 0.01, the Spearman’s test; Fig. 2c). Thus, spasticity and pain are associated with the activity-dependent boosting of synaptic excitation in sensory circuits. The changes were similar to those observed in the DH in neuropathic^21^ and chronic inflammatory pain conditions^22^.

The DH hyperexcitability originates from changes in both excitation and inhibition. Loss of inhibition within the DH accompanied abnormal pain sensation and chronic pain of different origins^23–25^. To test if this is the case in spasticity, we recorded inhibitory currents in lamina II interneurons. Disinhibition was manifested as an increase in the inter-event interval of sIPSCs (*p* < 0.001, the KS- and U-tests; Fig. 2d insert) – reflecting the decreased sIPSC frequency (by ~52%; Fig. 2e) – as a reduction in the sIPSC density (Fig. 2d), and as a drop in the current amplitude (from 22 pA in control to 18 pA in spasticity, decrease by ~18%, *p* < 0.001, U-test; Fig. 2e). The correlation profile of excitatory and inhibitory inputs received by individual interneurons further demonstrated boosted excitatory drive while reduced inhibitory control post-SCI (Fig. 2f).

### The SCI-induced changes in excitatory drive and postsynaptic AMPARs in specific types of DH interneurons

#### Heterogeneous excitatory drive to DH interneurons in control

**Figure 3:**
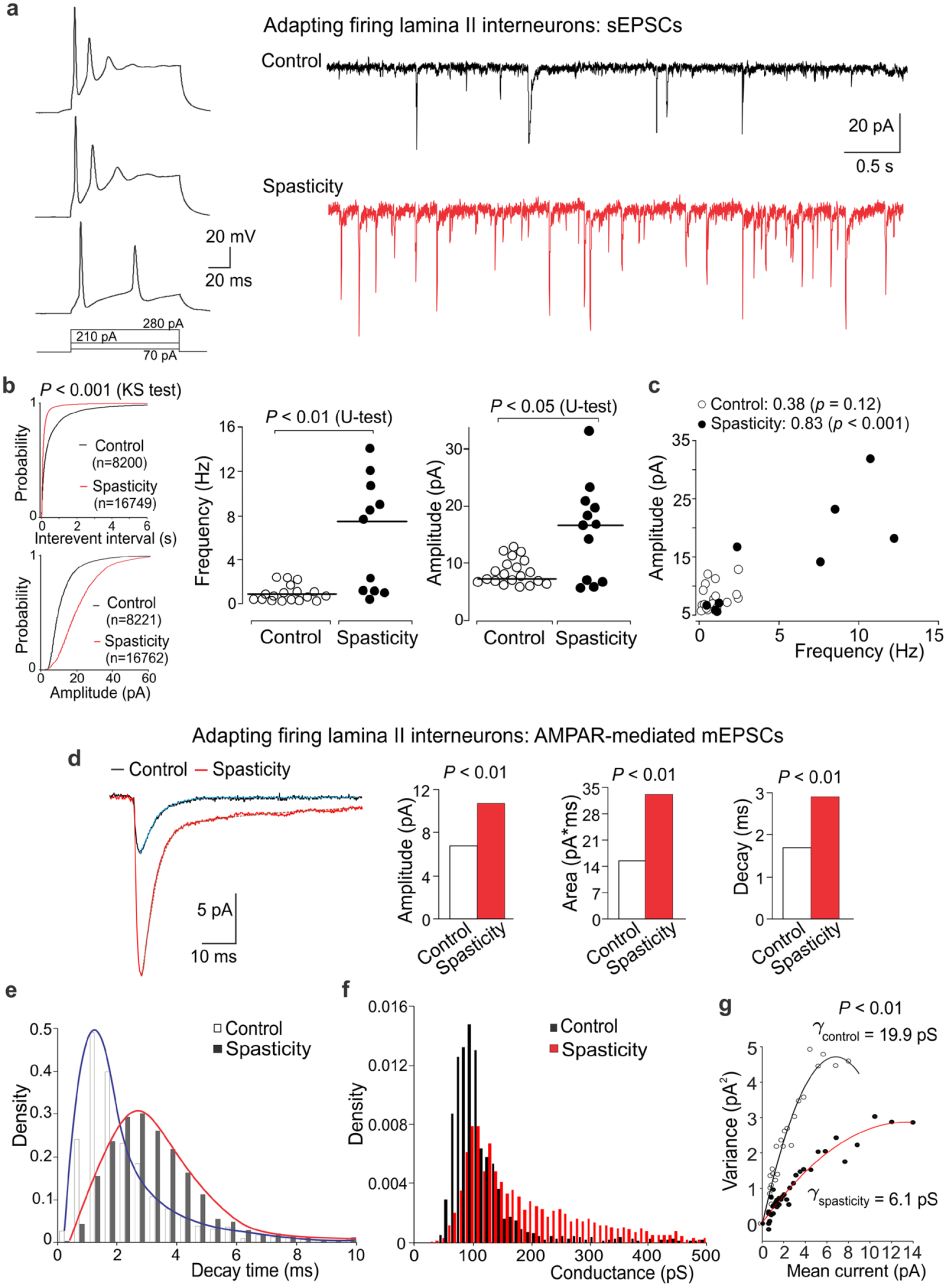
Boosted excitatory drive and upregulated postsynaptic AMPARs in AFNs post-SCI. (**a**) Examples of the current-clamp recordings of typical firing pattern of AFNs (left) in response to depolarizing currents (indicated on the bottom) and the voltage-clamp recordings of sEPSCs (Vh = −70 mV) in control and spastic animals (right). (**b**) Left, cumulative probability plots for the inter-event interval and the amplitude of sEPSCs in control and spasticity. Right, scatter plots of the median sEPSC frequency and the median current amplitude estimated for individual AFNs in control (n = 18 cells) and spasticity (n = 11 neurons). The Kolmogorov-Smirnov (KS) or the Mann-Whitney U-test significance is indicated. (**c**) The median sEPSC frequency plotted against the median current amplitude for individual AFNs in control and spasticity. The Spearman correlation coefficients with the Spearman’s non-parametric test significance are indicated. (**d**) The averaged AMPAR-mediated mEPSCs (left) and summary for the median current amplitude, area, and the decay kinetics (right) in control and spasticity. The Mann-Whitney U-test significance is indicated. (**e**,**f**) The probability density functions for the decay kinetics of postsynaptic AMPAR-mediated currents (**e**) and the estimated AMPAR-conductance (**f**) in AFNs in control and spasticity. (**g**) The single-channel conductance of postsynaptic AMPARs in AFNs in control (n = 6 cells, 453 currents) and spasticity (n = 4 cells, 670 currents). The bootstrap hypothesis test for two-sample problem is indicated.

**Figure 4:**
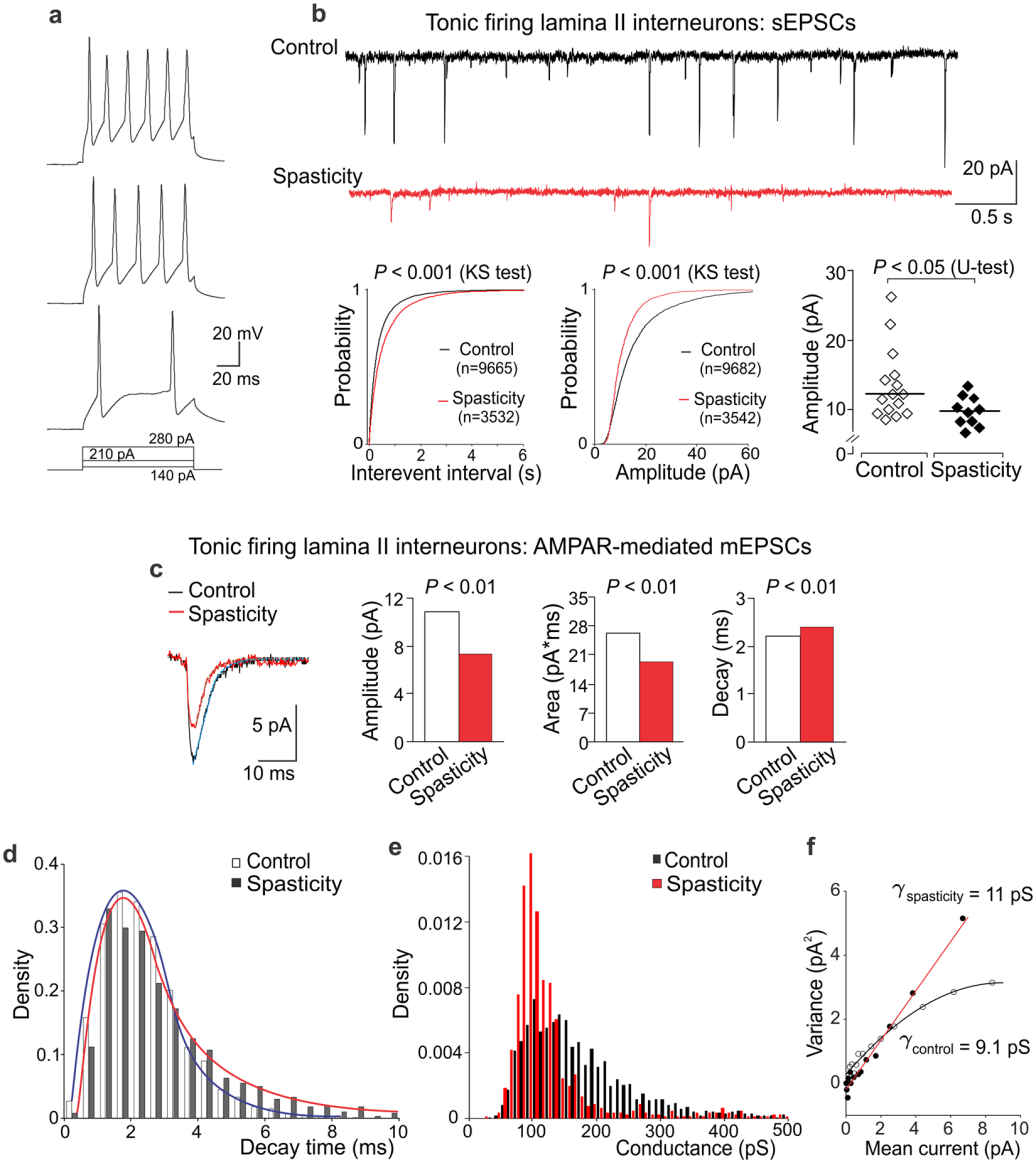
The reduced excitatory drive and diminished postsynaptic AMPAR-currents in TFNs post-SCI. (**a**) Examples of the current-clamp recordings of typical firing discharge of TFNs in response to depolarizing currents of increased intensity (indicated on the bottom). (**b**) Representative voltage-clamp recordings of sEPSCs (Vh = −70 mV) in control and spasticity (upper rows) and cumulative probability plots for the inter-event interval and the amplitude of sEPSCs (lower left) with the Kolmogorov-Smirnov (KS) test significance indicated. The scatter plots for the median current amplitude (lower right) in control and spasticity, estimated for each individual TFN (n = 15 cells in control and 10 cells in spasticity). The Mann-Whitney U-test significance is indicated. (**c**) The averaged AMPAR-mediated mEPSCs (left) and summary of the median current amplitude, the area, and the decay kinetics (right) in TFNs in control and spasticity. The Mann-Whitney U-test significance is indicated. (**d**,**e**) The probability density functions of the decay time of AMPAR-mediated currents (**d**) and the estimated AMPAR-conductance (**e**) in TFNs in control and spasticity. (**f**) The single-channel conductance of postsynaptic AMPARs in TFNs in control (n = 5 cells, 711 mEPSCs) and spasticity (n = 6 cells, 120 mEPSCs).

The interneuron-type-specific changes in excitatory DH transmission were reported in neuropathic^21^ and inflammatory pain conditions^22^. To test if the SCI-induced changes are uniform or different between types of interneurons, we examined two principal neuronal populations: AFNs (Fig. 3a) and TFNs (Fig. 4a), distinguishable by their electrophysiological properties. Consistent with our previous observations^22^, AFNs and TFNs revealed distinct excitatory drive in control (*p* < 0.001, the KS- and U-tests for the sEPSC inter-event interval and amplitude, data not shown). Changes in neuronal excitation post-SCI were also different; moreover, they were bidirectional.

#### Boosted drive and upregulated postsynaptic AMPARs in AFNs post-SCI

Excitatory drive was boosted to AFNs post-SCI. The inter-event interval of sEPSCs was decreased (up to 3-fold, *p* < 0.001, the KS-test), reflecting the increased frequency (from 0.83 Hz in control to 7.60 Hz in spasticity, increase in the median frequency up to 9-fold, *p* < 0.001, U-test; Fig. 3b). The median sEPSC amplitude was also increased (from 7.2 pA in control to 16.6 pA in spasticity, *p* < 0.05, U-test; Fig. 3b). Changes in both median frequency and amplitude (analysed for individual neurons) were correlated: the Spearman’s correlation coefficient was 0.83 in spasticity (n = 11, *p* < 0.001) *vs*. 0.38 in control (n = 17, *p* = 0.12, Spearman’s non-parametric test; Fig. 3c), implying the activity-dependent upregulation of receptors at glutamatergic synapses.

Postsynaptic AMPARs mediate the majority of glutamatergic transmission in sensory circuits^26^. For the assessment of the SCI-induced changes in postsynaptic AMPARs we recorded the AMPAR-mediated postsynaptic currents (see Methods). In AFNs, AMPARs were upregulated post-SCI, as revealed by the dramatically augmented current amplitude (Fig. 3d) and changes in other current parameters (Fig. S3). The median current amplitude was increased by ~60%, the current area by ~113%, the decay kinetics by ~71% (*p* < 0.001, U-test for either parameter; Fig. 3d). The pool of AMPAR-currents rearranged (Fig. 3e) such that proportion of the AMPAR-mediated currents with fast decay kinetics (≤2.5 ms) became reduced (*p* < 0.001, the KS-test), with a shift towards the AMPAR-currents with slower decay kinetics (>2.5 ms, *p* < 0.05, the KS-test). There were similar rearrangements in the pooled AMPAR-conductance: a drop in the density of AMPARs of smaller conductance along to the increased density of receptors of larger conductance (*p* < 0.001, the KS-test; Fig. 3f). Together it points to the AMPAR subunit re-composition at glutamatergic synapses post-SCI. Given that either slower desensitization or larger channel conductance characterizes the GluA2-containing AMPARs^27^, a drop in the density of both fast-decayed currents and smaller conductance indicates the loss of GluA2-lacking AMPARs. Consistently, the increased proportion of both AMPAR-currents of slower decay kinetics and larger channel conductance reflects the increased proportion of GluA2-containing AMPARs and suggests a promoted insertion of these receptors into glutamatergic synapses that is in line with the augmented current amplitude.

PS-NSFA revealed a drop in the single-channel AMPAR-conductance (γ): from 19.9 ± 3.7 pS (n = 6 AFNs, 453 AMPAR-currents) in control to 6.05 ± 1.32 pS (n = 4 cells, 670 AMPAR-currents) in spasticity (*p* < 0.001, bootstrap hypothesis test; Fig. 3g), confirming the lack of GluA2-lacking AMPARs from synapses of AFNs post-SCI.

#### Reduced excitatory drive and diminished AMPAR-currents in TFNs post-SCI

In contrast to AFNs, excitation of TFNs was reduced post-SCI. The inter-event interval of sEPSCs was increased (by ~49%, *p* < 0.001, the KS-test; Fig. 4b), reflecting the decreased frequency. The median current amplitude was also decreased (by ~20%, *p* < 0.05, U-test; Fig. 4b). This accompanied with the reduced AMPAR- current amplitude (Fig. 4c) and changes in other parameters (Fig. S4). The median current amplitude decreased by ~32% and the current area by ~26%; the decay kinetics prolonged by ~9% (*p* < 0.01, U-test for either parameter; Fig. 4c,d). Rearrangements in the AMPAR pool in TFNs were opposite to those in AFNs post-SCI. Whilst the density of fast-decayed currents remained unchanged (*p* = 0.72, the KS-test; Fig. 4d), the density of AMPAR-currents of smaller conductance increased in TFNs in spasticity (*p* < 0.001, the KS-test; Fig. 4e). The average γ also increased (from 9.1 ± 2.1 pS, n = 5 cells, 711 mEPSCs in control to 11 ± 2 pS, n = 6 cells, 120 mEPSCs in spasticity), though difference did not reach a significance with the bootstrap hypothesis test for two-sample problem (*p* = 0.67; Fig. 4f). Given that the AMPAR-current amplitude decreased in spasticity, such rearrangements may reflect a downregulation of postsynaptic AMPARs with a shift in the proportions of GluA2-containing to GluA2-lacking AMPARs.

Remarkably, γ was different between AFNs and TFNs in control: 19.9 ± 3.7 pS *vs*. 9.1 ± 2.1 pS, respectively (*p* < 0.01, bootstrap hypothesis test). A large difference in the conductance implies distinct receptor subunit composition between neuronal types that is consistent to heterogeneous synaptic drive^22^.

### The SCI-induced changes in synaptic inhibition in specific types of DH interneurons

#### Heterogeneous inhibitory control of DH interneurons in control

**Figure 5:**
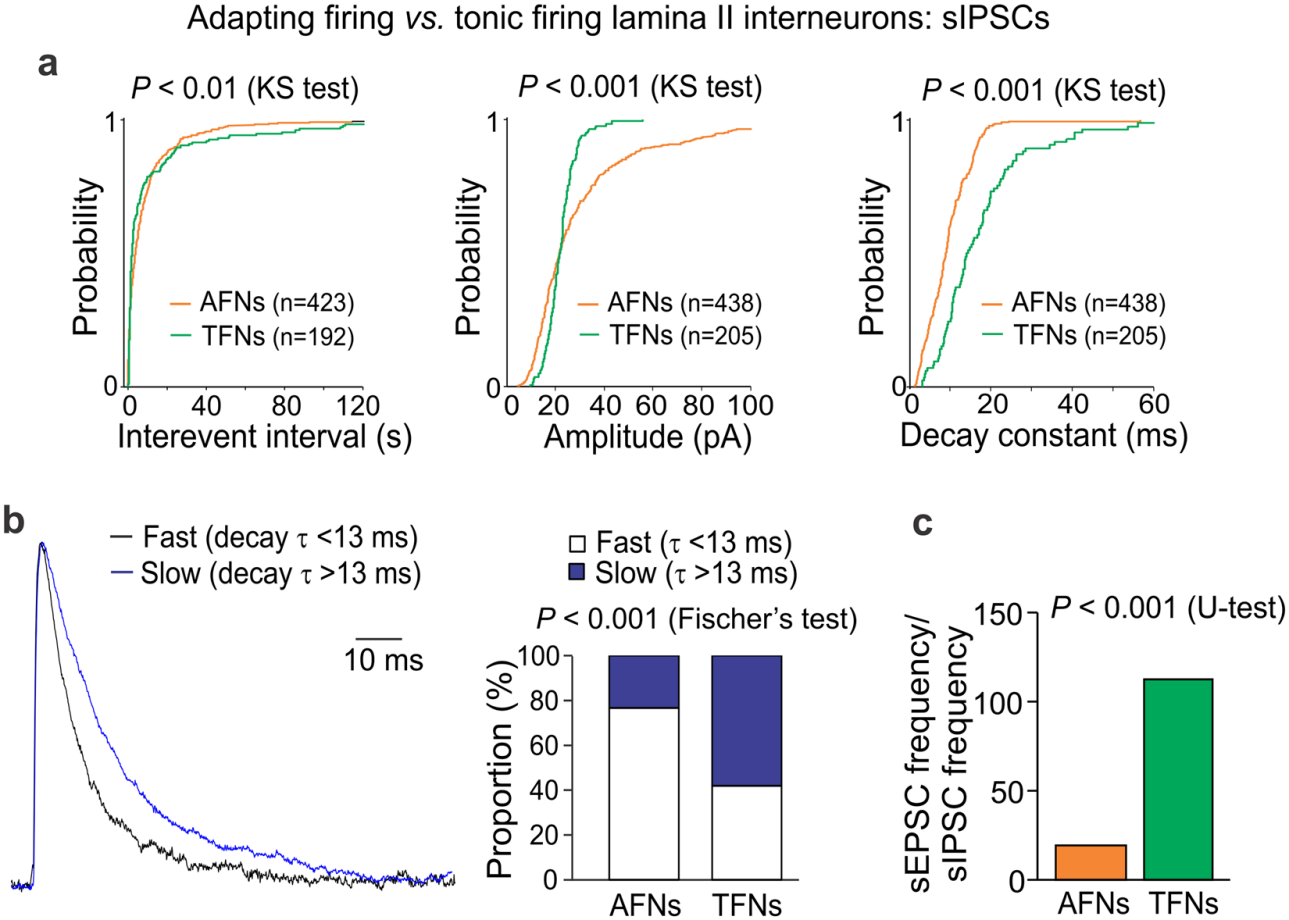
Heterogeneous inhibitory control of DH interneurons. (**a**) Cumulative probability plots for the inter-event interval, the amplitude and the decay constant of sIPSCs in AFNs and TFNs in control. The Kolmogorov-Smirnov (KS) test significance with the number of events for analysis is indicated. (**b**) Left, the fast sIPSCs (τ < 13 ms, black line) and slow sIPSCs (τ > 13 ms, blue line) in AFNs in control. The currents are average from 112 fast (τ = 10.5 ms) and 44 slow (τ = 19.6 ms) events recorded in 15 cells. Right, the relative proportions of fast to slow inhibitory currents in AFNs and TFNs in control. The Fisher’s exact test significance is indicated. (**c**) The average balance between neuronal excitation and inhibition, estimated as the ratio of the median sEPSC frequency to the median sIPSC frequency recorded from individual AFNs (n = 14 cells) or TFNs (n = 10 cells) in control. The Mann-Whitney U-test significance is indicated.

It emerges that inhibitory control is heterogeneous for distinct populations of DH interneurons. Therefore, we first examined synaptic inhibition of AFNs and TFNs in control. The parameters of sIPSCs were significantly different between neuronal types (*p* < 0.01, the KS-test for inter-event interval, *p* < 0.001, the KS-test for either current amplitude or decay; Fig. 5a). Analysis of the decay kinetics of sIPSCs unveiled a large difference in the proportion of fast (τ < 13 ms) to slow currents (τ > 13 ms) between neuronal types (*p* < 0.001, Fischer’s test; Fig. 5b), reflecting glycinergic and GABAergic inhibition, respectively [the characteristic decay time of glycine receptor (GlyR)-mediated postsynaptic currents in DH interneurons is 8.4 ms while 26.9 ms for the GABA receptor (GABAR)-mediated ones^25^]. The predominant fast sIPSCs [counted up to 77% of the entire pool of inhibitory events in AFNs] indicate that AFNs receive major glycinergic inhibitory control, with much less GABAergic inhibition [slow sIPSCs counted ~23%]. In contrast, TFNs demonstrated prevailing GABAergic inhibition (58%) balanced with glycinergic one (42%). Our data are consistent with two distinct populations of glycine- or GABA-dominant DH interneurons in lamina I-III^25^.

Having observed the difference in both excitatory drive and inhibitory control of DH interneurons, we then estimated the relative balance between synaptic excitation and inhibition that neurons receive simultaneously (see Methods for details). The ratio of the median sEPSC frequency to the median sIPSC frequency was 19.8 (n = 14) for AFNs but 113.0 (n = 10) for TFNs (*p* < 0.001, U-test; Fig. 5c), demonstrating a moderate synaptic drive to excitatory interneurons while boosted excitatory drive to inhibitory interneurons in control.

As in the case of synaptic excitation, changes in neuronal inhibition post-SCI were neuronal-type-specific; moreover, they were opposite to excitatory ones.

#### The SCI-induced disinhibition of AFNs

**Figure 6:**
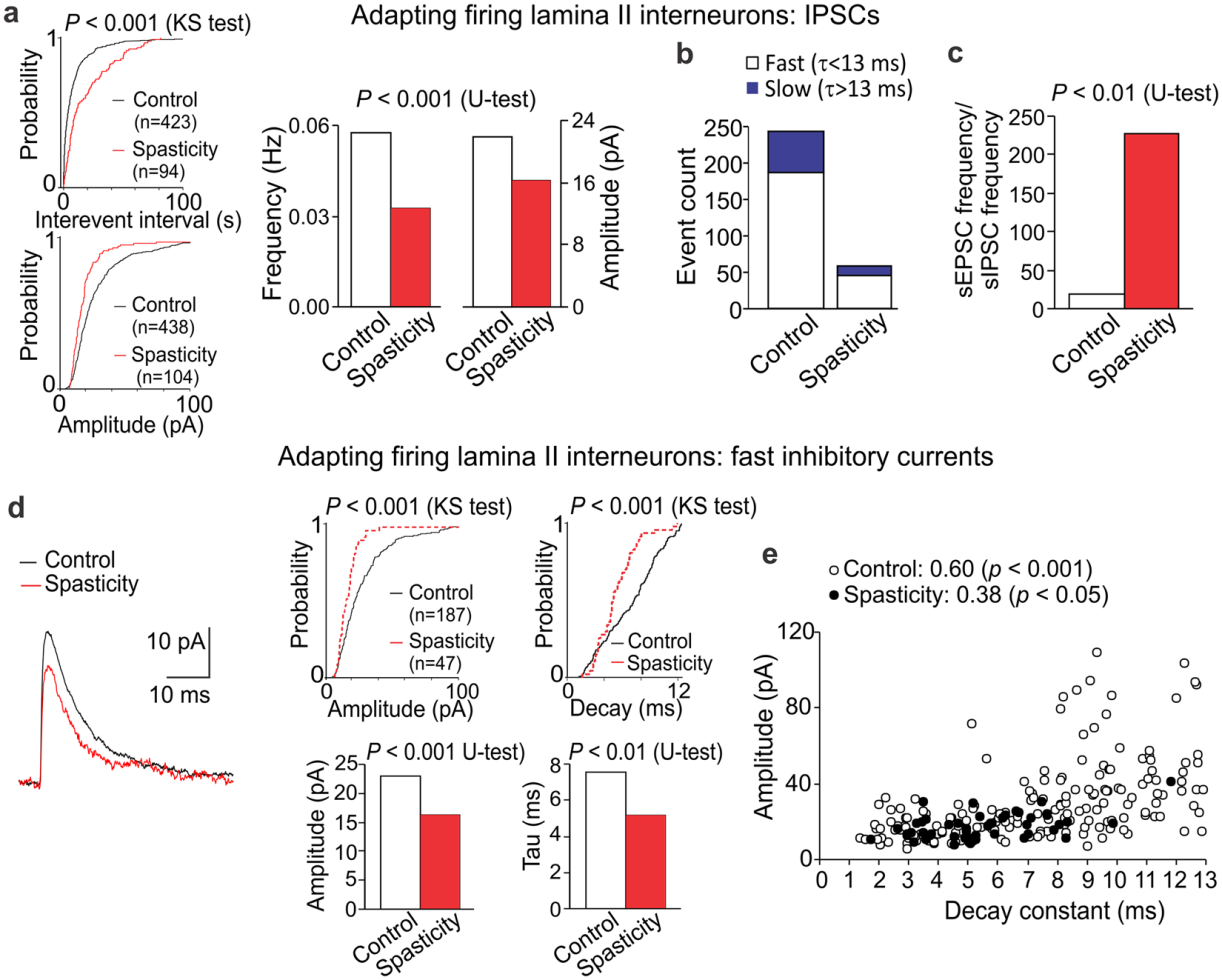
The SCI-induced loss of inhibition in AFNs. (**a**) Cumulative probability plots for the inter-event interval and the amplitude of sIPSCs (left) and summary of the median frequency and the median current amplitude (right) in AFNs in control and spasticity. (**b**) Occurrence of the fast (τ < 13 ms) and the slow (τ > 13 ms) inhibitory currents in different experimental conditions. (**c**) The average balance between synaptic excitation and inhibition of AFNs, estimated as the median frequency of sEPSCs to the median frequency of sIPSCs for each individual AFN in control (n = 14 cells) and spasticity (n = 10 cells). (**d**) Left, examples of the fast inhibitory currents in control (black line, average of 112 fast currents recorded in 15 AFNs) and in spasticity (red line, average of 25 fast currents recorded in 10 AFNs). Right, cumulative probability plots (upper raw) and summary of the median current amplitude and the decay time constant (lower raw) in AFNs in control and spasticity. (**e**) The amplitude of fast IPSCs (τ < 13 ms) plotted against its current decay constant in AFNs in control and spasticity. The Spearman correlation coefficients and the Spearman’s non-parametric test significance are indicated. The Kolmogorov-Smirnov (KS) or the Mann-Whitney U-test significance is indicated.

There was a disinhibition of AFNs post-SCI, manifested as an increase in the inter-event interval of sIPSCs (*p* < 0.001, the KS- and U-tests) – reflecting the decreased sIPSC frequency (by ~43%) – and as a decrease in the current amplitude (by ~25%, *p* < 0.001, the KS- and U-tests; Fig. 6a). GABAergic and glycinergic inhibition were both dramatically reduced in AFNs (up to 4-fold; Fig. 6b) and the slow [GABAR-mediated] inhibitory currents became rarely detectable post-SCI. The median amplitude of fast [GlyR-mediated] inhibitory currents dropped (from 23 pA in control to 16 pA in spasticity, *p* < 0.001, the KS- and U-tests) and the current decay became faster (7.6 ms in control *vs*. 5.2 ms in spasticity, *p* < 0.01, U-test; Fig. 6d). The changes correlated (*p* < 0.05, the Fisher r-to-z transformation compared to control): the Spearman’s correlation coefficient was 0.38 in spasticity (*p* < 0.05, Spearman’s non-parametric test) and 0.60 in control (*p* < 0.01, Spearman’s non-parametric test; Fig. 6e). Besides, we observed the lack of currents of large amplitude (above 30 pA) and slower decay kinetics (8–13 ms).

The SCI-induced changes in both synaptic excitation and inhibition resulted in largely amplified excitability of AFNs (above 11-fold): the balance shifted from 19.8 (n = 14) in control to 228.2 (n = 10, *p* < 0.01, U-test) in spasticity (Fig. 6c).

#### The SCI-induced reshuffle in GABAergic and glycinergic inhibition of TFNs

**Figure 7:**
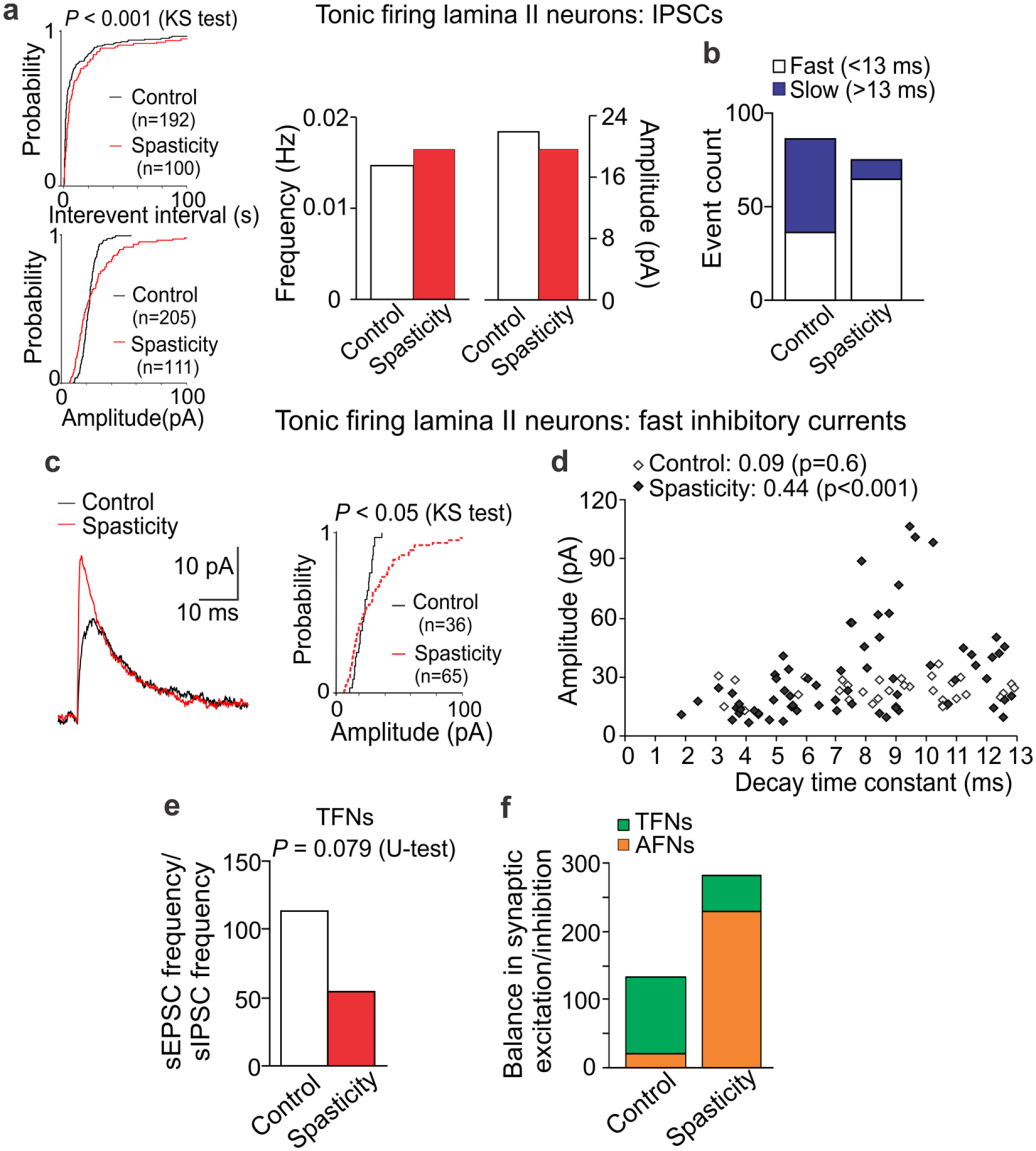
The SCI-induced reshuffle between GABAergic and glycinergic inhibition in TFNs. (**a**) Cumulative probability plots for the inter-event interval and the amplitude of sIPSCs (left) and summary of the median frequency and the median current amplitude (right) in TFNs in control and spasticity. (**b**) Occurrence of the fast (τ < 13 ms) and the slow (τ > 13 ms) inhibitory currents in TFNs in control and spasticity. (**c**) Examples of the fast inhibitory currents (left) and cumulative probability plots for the current amplitude (right) in TFNs in control and spasticity. The currents are average from 25 fast sIPSCs recorded in 11 TFNs in control (black line) and 53 fast sIPSCs recorded in 11 cells in spastic animals (red line). (**d**) The amplitude of fast sIPSCs plotted against the correspondent decay constant in TFNs from control and spastic animals. The Spearman correlation coefficients with the Spearman’s non-parametric test significance are indicated. (**e**) The average balance between synaptic excitation and inhibition of TFNs, estimated as the median frequency of sEPSCs to the median frequency of sIPSCs for each individual TFN in control (n = 10 cells) and spasticity (n = 9 cells). (**f**) The relative balance between excitatory drive and synaptic inhibition of AFNs and TFNs in control and post-SCI. The Kolmogorov-Smirnov (KS) or the Mann-Whitney U-test significance is indicated.

Neither median frequency (*p* = 0.8) nor median amplitude of sIPSCs (*p* = 0.4, U-test) was changed in TFNs post-SCI, though cumulative distributions revealed a significant difference between experimental conditions (*p* < 0.001, the KS-test; Fig. 7a). Analysis of the fast and slow inhibitory currents unveiled their rearranged proportion: a drop in the slow [GABAR-mediated] currents (from 58% in control to 13% in spasticity, *p* < 0.001, Fischer’s test) while an opposite increase in the fast [GlyR-mediated] currents (from 42% in control to 87% in spasticity, *p* < 0.001, Fischer test; Fig. 7b). Thus, inhibitory control of TFNs reshuffled post-SCI by eliminating GABAergic whilst potentiating glycinergic inhibition. Such potentiation was associated with the increased amplitude of GlyR-currents (*p* < 0.05, the KS test; Fig. 7c) correlated with the prolonged current decay kinetics: the Spearman’s rank correlation coefficient was 0.44 in spasticity (*p* < 0.001, Spearman’s non-parametric test) and 0.09 in control (*p* = 0.6, Spearman’s non-parametric test; Fig. 7d). The correlation profile (Fig. 7d) revealed appearance of fast currents of large amplitude (40–120 pA) and slower decay kinetics (7–13 ms) that suggests GlyR upregulation at inhibitory synapses post-SCI.

Potentiated synaptic inhibition of TFNs together with the reduced AMPAR-mediated excitation resulted in suppressing of TFNs (up to 2-times): the balance dropped from 113 (n = 10) in control to 54.6 (n = 9) in spasticity (*p* = 0.079, U-test; Fig. 7e). The overall balance between excitation and inhibition within the DH was re-directed from driving of inhibitory interneurons in control to boosting of excitatory interneurons post-SCI (Fig. 7f).

## Discussion

This is the first study of central mechanisms underlying chronic pain associated with spasticity that reveals the SCI-induced shifts in neuronal excitation and inhibition resulted in the DH circuit hyperexcitability. We provide here quantitative counting of synaptic excitation and inhibition in the DH lamina II interneurons, demonstrating the reciprocal changes occurring in the interneuron-type-specific manner to elucidate how nociceptive processing changes in both spasticity and pain, the most challenging complications post-SCI.

A high occurrence of chronic pain after SCI (up to 80% cases) has been reported by clinical studies^5, 6^. This pain could develop through diverse clinical features with a low capability to be cured. In animal studies, the SCI-induced nociceptive hypersensitivity was correlated with severity of injury^18, 28^. In our modelling of unilateral SCI, animals displayed severe motor deficit that exceeded on average the level 1 on the Ashworth rating scale and lowered the level 7 on the BBB scale. Recordings of the H-reflex, a physiological marker of spasticity^3, 29^, revealed the increased ratio of H/M-wave responses in the SCI-injured animals, evidencing the hyperexcitability within sensorimotor pathways. Consistently, animals exhibited pain hypersensitivity in response to thermal stimulation of hindpaw (both ipsilateral and contralateral) detected with a semi-automated method of measuring the thermal threshold^22, 30, 31^. The hypersensitivity persisted over weeks post-SCI, evidencing chronic pain, which correlated with the degree of spasticity (the H/M ratio). This pain can exacerbate to unceasing chronic pain and become intolerable, as suggested here by a self-mutilatory behaviour in some of the rats, which has been proposed as a readout of neuropathic pain^17, 20^. However, our plantar testing with von Frey monofilaments failed to directly demonstrate the mechanical hyperalgesia and allodynia post-SCI that very likely relates to a restricted motility of hindlimbs in the SCI-injured animals since applications of the classical behavioural methodology for studying tactile sensation are less accurate or impractical in animals with severe motor dysfunction and limited weight support^19, 28^.

The present study focuses on central mechanisms of chronic pain and permanent changes in sensory input integration in the DH for pain processing. To achieve this, patch-clamping of lamina II interneurons, which process input integration from both primary sensory afferents and other interneurons, was performed in the spinal cord below lesion from the SCI-injured animals experiencing long-term spasticity (1–2-month) that yields benefits of the direct recordings from vulnerable DH interneurons (2–3-month-old) following chronic pathology. The present study demonstrates the DH hyperexcitability in conditions of both spasticity and pain post-SCI. Although we cannot rule out that the DH hyperexcitability exaggerated with a tissue damage produced by surgical procedure as no sham-operated control was tested electrophysiologically, the DH hyperexcitability post-SCI has been emerged from the hyperexcitation of the dorsal root ganglion neurons and the augmented primary afferent inputs^9^, and the increased interneuron firing in response to noxious or innocuous stimuli^15, 32^, arguing for both peripheral and central sensitization below and above lesion causing aberrant pain processing. Our recordings of excitatory and inhibitory currents in lamina II interneurons demonstrate that the DH circuit hyperexcitability results from two mechanisms – boosted excitation and synaptic disinhibition – those contribute to the hyperexcitability of the DH post-SCI. Most attention through studies of chronic pain of different origins has hitherto been given to the loss of GABAergic inhibition^23, 33, 34^, with a possible death of GABAergic interneurons^35^. A shift in chloride gradient due to a downregulation of the chloride transporter after SCI has been also demonstrated to reverse potential for GABA^16, 36^, a mechanism attenuating GABAergic inhibition in DH interneurons^37^.

Central to this study is the interneuron-type-specific changes in synaptic excitation and inhibition, occurring in the bidirectional and reciprocal manners in two principal types of DH interneurons, AFNs and TFNs, those differ by electrophysiological properties^38–40^, synaptic drive^22, 41^, and neurochemical phenotype^39^. A moderate synaptic drive to excitatory AFNs in control has been boosted following SCI, opposite to a promoted excitatory drive to inhibitory TFNs in control, which has been reduced after SCI. These changes were similar to those in chronic inflammatory pain^22^, arguing for similar mechanisms of chronic pain. Boosting of AFNs was mirrored by the activity-dependent upregulation of postsynaptic AMPARs, though the AMPAR-mediated currents were decelerated and the single-channel conductance was reduced post-SCI. The slower decay kinetics, a prominent characteristics of GluA2-containing AMPARs^42, 43^, may reflect a promoted insertion of GluA2-containing AMPARs into glutamatergic synapses – coherent with the augmented current amplitude – while a drop in the single-channel conductance very likely relates to a loss of GluA2-lacking AMPARs (only fast AMPAR-currents [decay from 1 to 5 ms] were analyzed). The rearranged pool of postsynaptic AMPAR-currents indicates the AMPAR subunit re-composition post-SCI, with a few mechanisms involved whose biophysical and molecular basis remain to be elucidated. Changes in TFNs (e.g., the decreased AMPAR-current amplitude, rearranged pools of the decay kinetics and the single-channel conductance) were opposite to AFNs, indicating differential mechanisms of the AMPAR subunit re-composition between neuronal types. The decreased amplitude may relate to internalization of GluA2-containing AMPARs, demonstrated for the synapses between primary afferents and DH interneurons in chronic inflammatory pain conditions^44^, although TFNs lost the GluA2-lacking AMPARs in the peripheral injury-induced neuropathic pain^21^. Remarkably, the single-channel conductance of postsynaptic AMPARs was different between AFNs and TFNs even in physiological conditions (19.9 pS *vs*. 9.1 pS, respectively) that further proves a distinct receptor subunit composition and is consistent to the distinct synaptic drive to AFNs and TFNs^22^ and to the higher surface expression of GluR1 in inhibitory DH interneurons while GluR2 in excitatory ones^45, 46^.

The present study stresses the SCI-induced shifts in synaptic excitation and inhibition occurring in the bidirectional and reciprocal manner in two principal types of sensory interneurons, excitatory and inhibitory, such that changes in neuronal inhibition opposed changes in their excitation. Such contraposition resulted in a shifted balance of neuronal excitation and inhibition, suggesting a phenomenon of the reciprocal reshuffle between excitatory and inhibitory transmission as a neurophysiological mechanism of amplifying nociceptive processing. The SCI-induced disinhibition of AFNs (major excitatory interneurons) through diminishing of both GABAergic and glycinergic controls contrasted with inhibition of TFNs (inhibitory interneurons) despite of their loss of GABAergic control post-SCI. Whilst loss of GABAergic inhibition is abundantly evidenced in the DH after SCI^16, 34, 37^, a reshuffle between GABAergic and glycinergic inhibition has not been to the date reported. Potentiation at glycinergic synapses on inhibitory interneurons would promote their inhibition, retard inhibitory control of sensory circuits, and cause the DH disinhibition^47^. Notably, the fast inhibitory currents became often in TFNs post-SCI, with larger amplitude and slower decay kinetics that may reflect GlyR upregulation, though changes in biophysical properties of the receptors and/or the number of functional synapses cannot be also excluded. The SCI-induced potentiation of glycinergic inhibition at inhibitory synapses together with the reduced AMPAR-mediated excitation at glutamatergic synapses represent two coherent mechanisms of reciprocal retardation of inhibitory interneurons for circuitry disinhibition and central sensitization mediating chronic pain.

The bidirectional and opposite changes in excitatory and inhibitory transmission in two types of interneurons underlyi the DH hyperexcitability post-SCI that could, in turn, contribute to hyperexcitability of the ventral horn. This may include interconnections between the lamina II interneurons with interneurons of deeper laminae, e.g. involving the laminae IV–V interneurons projecting ipsilaterally to motoneurons of the ventral horn^48^. Whilst we did not attempt to focus on components of the motor circuit control, extensive studies over years demonstrated the reduced intrinsic excitability of motoneurons with normalization of uncontrolled circuit function following pharmacological targeting of sensory transmission to motoneurons^49, 50^ or electrical stimulation of spinal circuitry^51, 52^ that ultimately improved management of spasticity post-SCI. This is also consistent to the microsurgical dorsal root entry zone (DREZ)-tomy relief in neurogenic pain and hyperspastic states in patients with severe spasticity and pain, implemented in clinics over decades ago^53^. Although the nature of spasticity is complex and not finally explained, we provide here a central mechanism of unbalancing the sensory circuit component relying on the bidirectional and reciprocal changes in excitatory and inhibitory transmission in DH interneurons in conditions of spasticity and pain, the most severe complications post-SCI.

## Methods

All animal procedures were approved by the Animal Ethics Committee in Bogomoletz Institute of Physiology and were in accordance with the European Commission Directive (86/609/EEC). All efforts were made to avoid or minimize animal suffering and to reduce the number of animals used.

### Experimental SCI

For modelling of experimental SCI hemisection of the spinal cord was used in 21–25-day-old male Wistar rats. For the surgery a rat was deeply anesthetized with ketamine and xylazine (70 mg/kg and 15 mg/kg, respectively, intraperitoneal injection), confirmed with the lack of the corneal reflex. After surgical area was prepared and treated with antiseptics, one longitudinal skin incision was made around Th_8_–L_2_ segments and muscles were retracted to expose the spinal column. Laminectomy was carried out at Th_10_–Th_11_ segments, pre-determined by palpation of the dorsal surface and by counting spinous processes to locate the laminae. Hemisection of the spinal cord was performed transversally at Th_11_ on the left side, with extra care taken to avoid any damage to the dorsal vessel or its branches (Fig. 1a). To ensure that hemisection carried out completely, one branch of ophthalmologic tweezers was pulled through the spinal cord incision. The muscles and fascia were sutured and wound closed. Animals received postoperatively bicillin (1 million U/kg) and dexamethazon (6 mg/kg). Post-hoc histology confirmed hemisection not crossing the midline in transverse spinal cord sections. Sham surgery was performed by exposing the spinal cord to a similar procedure without hemisection.

Only animals revealing motor deficit ipsilateral to the hemisection side were included into the study; in case of sparse animals revealed neurological deficit on the contralateral side post-SCI, they were discarded. We also discarded animals if their scoring exceeded the level 7 on the Basso, Beattie, and Bresnahan (BBB) rating scale and was level 0 on the Ashworth scale on day 2–3 post-lesion (less than 15% animals).

### Behavioural assessments post-SCI

For the assessment of motor deficit and muscle tone of hindlimb post-SCI we used behavioural tests – the BBB and the Ashworth rating scales, respectively. For the assessment of pain hypersensitivity of each hindlimb post-SCI we utilized a semi-detected method of measuring the latency of paw withdrawal (the Hargreaves technique) and the method of von Frey monofilaments (Extended Experimental Procedures). Animals were also monitored for a self-mutilatory behaviour (Extended Experimental Procedures), which has been proposed as a readout of neuropathic pain in animal studies^17, 20^. Behavioural assessments were performed in a quiet room by the same experimenter for a given test.

#### The BBB rating scale

The BBB scale was used for the assessment of motor function of hindlimbs in each animal. The test is widely utilized for evaluation of animal locomotion, with some modifications. Our scoring was based on the BBB rating scale for the incompletely spinal cord injury in rats^54, 55^. Tested animal was allowed to freely move and evaluated for its hindlimb joint movements, paw placement, weight support, and limb coordination according to the scoring criteria described in details in Table 1. Each animal was evaluated before lesion, on day 2–3 post-lesion and then consistently scored for at least 3 months postoperatively. Only scores of hindlimb on the hemisected side (left side) are presented.

#### The Ashworth scale

The Ashworth rating scale was used for the assessment of muscle tone and hindlimb rigid post-SCI according to the adapted Ashworth scale for rats^56, 57^. We evaluated positioning of hindlimb ipsilateral to the hemisected side as described in details in Table 1. Each animal was evaluated before lesion, on day 2–3 post-lesion and then for at least 3 months postoperatively.

#### Hargreaves plantar test for thermal pain hypersensitivity

Peripheral sensitivity to the thermal (heat) stimulus was measured in rats with Hargreaves technique as described previously^30, 31, 58^. Briefly, after an animal habituated to the Plexiglas chamber located above a light box (Ugo Basile Model 7370 Plantar Test), a radiant heat was applied to the middle of the plantar surface of one hind paw. The light beam was automatically turned off when animal lifted its paw. Trial was repeated 3–5 times with an interval between measurements 3 to 5 minutes. The time between the start of stimulus and animal lifted its paw, the withdrawal latency, was measured, which represents the thermal nociceptive threshold.

### Hoffman (H)-reflex measurement

The H-reflex measurements were performed using a multimodality stimulator system (Neurosoft, Russia) as described in details elsewhere^29^. After a rat was anaesthetized, the longitudinal incision was made along the femoral surface of a tested hindlimb to expose sciatic nerve. Stimulating bipolar hook electrode was placed on the nerve surroundings; recording needle electrode was placed into the gastrocnemius muscle. The nerve was stimulated using square pulses (1–2 mA, 5-ms duration, 0.2 Hz) of increased stimulus intensity (1-mA-increment). Several trials (5 sweeps/trial) were recorded with at least 5-min-rest intervals between trials. The M- and the H-waveform peak amplitudes were measured and the ratio of H- to M-wave amplitudes was calculated. In the case of the end of M-wave and the beginning of H-wave overlapped, the M- and the H-wave currents were summated since the M-wave amplitude and the latency remained stable, not affecting the H-wave amplitude comparison^29^. The H-reflex measurements were performed at the time period of 4–5 weeks postoperatively.

### Spinal cord slice preparation

Spinal cord slices were prepared from 2–3-month-old rats as described previously, with minor modifications^22, 38^. The spinal cord below hemisection (lumbar segments) was removed and placed in an ice-cold dissection solution that contained (in mM) 250 sucrose, 2 KCl, 1.2 NaH_2_PO_4_, 0.5 CaCl_2_, 7 MgCl_2_, 26 NaHCO_3_, 11 glucose, oxygenated with 95% O_2_ and 5% CO_2_. Transverse slices (350–400 μm thickness) were cut with a HA752 vibratome (Campden Instruments, Loughborough, UK) and maintained in a physiologic Krebs solution that contained (in mM) 125 NaCl, 2.5 KCl, 1.25 NaH_2_PO_4_, 2 CaCl_2_, 1 MgCl_2_, 26 NaHCO_3_, 10 glucose, oxygenated with 95% O_2_ and 5% CO_2_.

### Electrophysiology

Whole-cell recordings were made from lamina II interneurons ipsilateral to hemisection, using a Multipatch 700B amplifier controlled with pClamp 9.2 software (Molecular Devices, USA). Neurons were visualized with infrared optics using a 60× water-immersion objective on an Olympus BX50WI upright microscope (Olympus, Japan). Recording pipettes (resistance 4–5 MΩ) were filled with an internal solution containing (in mM) 133 K-gluconate, 5 NaCl, 0.5 MgCl_2_, 10 HEPES-Na, 2 MgATP, 0.1 GTP-Na, 0.5 EGTA (pH 7.2, osmolarity 290 mOsM). Interneurons were categorized according to their discharge patterns in response to the series of depolarizing current of 0.5–1-s duration^22, 30, 38^. Tonic-firing, adapting-firing, transient (initial burst)-firing, and single-spiking discharge patterns were recorded. Because all these patterns except the tonic-firing are adapting due to the A-type potassium-current-related pattern of discharge, we grouped them together that neurons were divided in two groups: “adapting-firing” (AFN) and “tonic-firing” neurons (TFN)^22, 30, 38^. TFNs were defined as those able to support continuous firing during depolarizing current and increase firing frequency with increasing the stimulus intensity (Fig. 4A); this neuronal type represents putative inhibitory interneurons^39, 40^.

Spontaneous excitatory postsynaptic currents (sEPSCs) were recorded in interneurons at –70 mV for at least 5 minutes. The membrane potential was then switched to 0 mV to record spontaneous inhibitory postsynaptic currents (sIPSCs) from the same neuron for the next 5 minutes (after baseline stabilization at 0 mV)^22^. To isolate the AMPA receptor (AMPAR)-mediated component, miniature EPSCs (mEPSCs) were recorded at –70 mV in the continuous presence of a cocktail of antagonists, which consisted of TTX (200 nM), APV (50 µM), bicuculline (5 µM), strychnine (2 µM), CdCl_2_ (100 µM), administered 10 min prior to the recording. Only neurons with a stable baseline during the entire recording were included into analysis.

### Data analysis

Mini Analysis Program (Synaptosoft, Decatur, GA) was used for analysis as described previously^22^. The kinetics of EPSCs were analysed within the time frame of 10% to 90% of current for rise time and 100% to 37% of current for decay time. The area of currents was calculated as an integral within the time frame of current initiation until 37% of current decay. The decay kinetics of sIPSCs (τ) was calculated within the time frame of 100% to 37% of current decay fitted with the mono-exponential function. The balance between synaptic excitation and inhibition received by individual neurons simultaneously was estimated as the ratio of excitatory inputs [the median sEPSC frequency] to inhibitory inputs [the median sIPSC frequency] recorded in the same neurons. The AMPAR-conductance was calculated according to the following equation:

$${\rm{G}}={{\rm{I}}}_{{\rm{\max }}}/({{\rm{V}}}_{{\rm{rev}}}-{{\rm{V}}}_{{\rm{m}}})$$

where I_max_ is the peak of AMPAR-mediated current, V_rev_ is the reversal potential, V_m_ is the membrane potential.

The data sets were probed for normality using the Shapiro-Wilk test. Data sets not normally distributed were compared using a nonparametric Mann-Whitney U-test and results were presented as medians with interquartile ranges (IQR). The Kolmogorov-Smirnov two-sample test (KS-test) was used to compare the distributions in parameters between groups. Correlations between variable, non-parametrically distributed parameters were assessed using a Spearman non-parametric rank correlation. The Fisher exact test was used to determine statistical difference between two categorical variables. A *P* value less than 0.05 was considered as statistically significant for either test.

### Peak-scaled non-stationary fluctuation analysis

The single-channel conductance (γ) of postsynaptic AMPARs was estimated using the peak-scaled non-stationary fluctuation analysis (PS-NSFA)^59^. Only AMPAR-mediated mEPSCs with the decay time from 1 to 5 ms were used for the analysis if displayed a stable baseline with no overlapping events during decay. Selected currents were averaged to obtain the mean mEPSC and then individual currents were scaled to the mean to obtain the peak scaled current according to the Equation (1)

1 $${I}_{i}^{peak-scaled}={I}_{i}-\langle I\rangle \frac{{\rm{\max }}({I}_{i})}{{\rm{\max }}\,\langle I\rangle },$$

where *I*_*i*_ is an individual current, 〈*I*〉 is the mean mEPSC.

Variance of the peak-scaled mEPSCs, ${\sigma }^{2}({I}^{peak-scaled})$, was plotted versus mean and fitted with parabola described by the Equation (2), using the weighted least squares method with weights ${\omega }^{2}=\frac{1}{variance({\sigma }_{i}^{2})}$.

2 $${\sigma }^{2}({I}^{peak-scaled})={i}_{ch}\langle I\rangle -\frac{{\langle I\rangle }^{2}}{N}+{\sigma }_{0}^{2},$$

where 〈*I*〉 is the mean mEPSC, *N* is the functional number of channels, ${\sigma }_{0}^{2}$ is the variance of background noise and *i*_*ch*_ is the single-channel current. Finally, γ was calculated from the equation

3 $$\gamma =\frac{{i}_{ch}}{V-{E}_{rev}},$$

where *V* is the holding potential, *E*_*rev*_ is the reversal potential (0 mV).

The accuracy of the estimated γ was calculated using bootstrap method where 1000 bootstrap samples were generated for each group; significant difference was assessed with the bootstrap hypothesis test for two-sample problem.

## Electronic supplementary material

Supplementary Information
