# Supplementary material for: Opposite, bidirectional shifts in excitation and inhibition in specific types of dorsal horn interneurons are associated with spasticity and pain post-SCI
Source: Sci Rep. 2017 Jul 19;7:5884. doi: 10.1038/s41598-017-06049-7 (PMC5517549; doi:10.1038/s41598-017-06049-7)
Supplement: Supplementary file 1 — Supplementary Information [file 41598_2017_6049_MOESM1_ESM.doc]

**SupplementaRY information**

to the article

**“Opposite, bidirectional** **shifts in excitation and inhibition in specific types of dorsal horn interneurons are associated with spasticity and pain post-SCI”**

by Olga Kopach, Volodymyr Medvediev, Volodymyr Krotov, Anya Borisyuk, Vitaliy Tsymbaliuk, Nana Voitenko

**
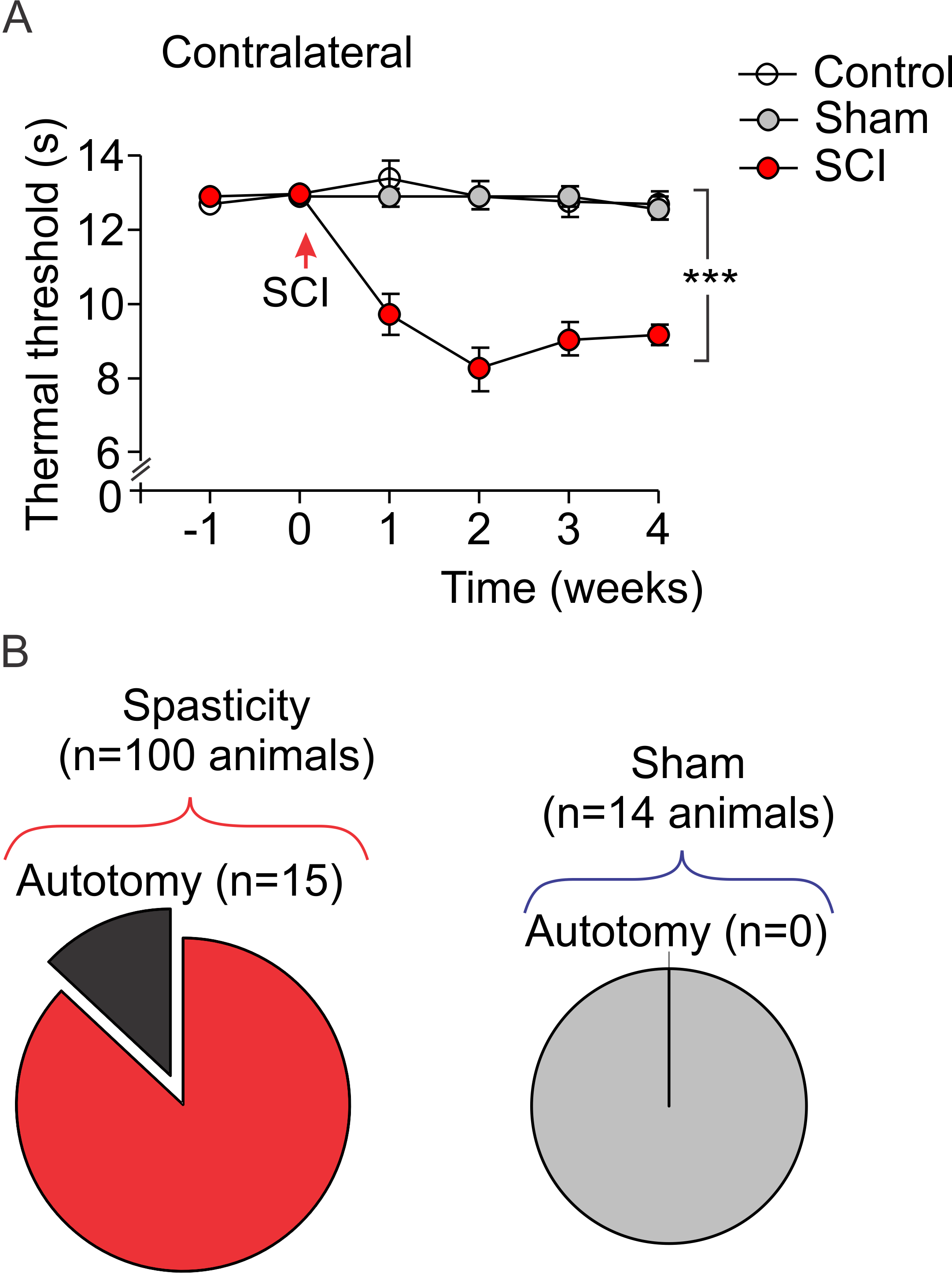
**

**Figure S1.** SCI results in the peripheral thermal pain hypersensitivity on the contralateral to hemisection side, related to Figure 1.

1. The time course of changes in the thermal nociceptive threshold on the contralateral hind paw in different experimental groups, demonstrating the long-lasting pain hypersensitivity in the SCI-animals postoperatively (n = 11 control rats, n = 14 sham-operated rats and n = 15 animals post-SCI). ****P* < 0.001 (ANOVA with Bonferroni post-hoc test). Data are expressed as mean; error bars, SEM.
2. Occurrence of the self-mutilatory behaviour (autotomy) postoperatively revealing experiencing of intolerable chronic pain by the SCI-injured animals (left, n = 15 animals displaying autotomy out of 100 of the pooled SCI-group) but not the sham-operated group (n = 0 with autotomy out of 14 sham-operated rats).


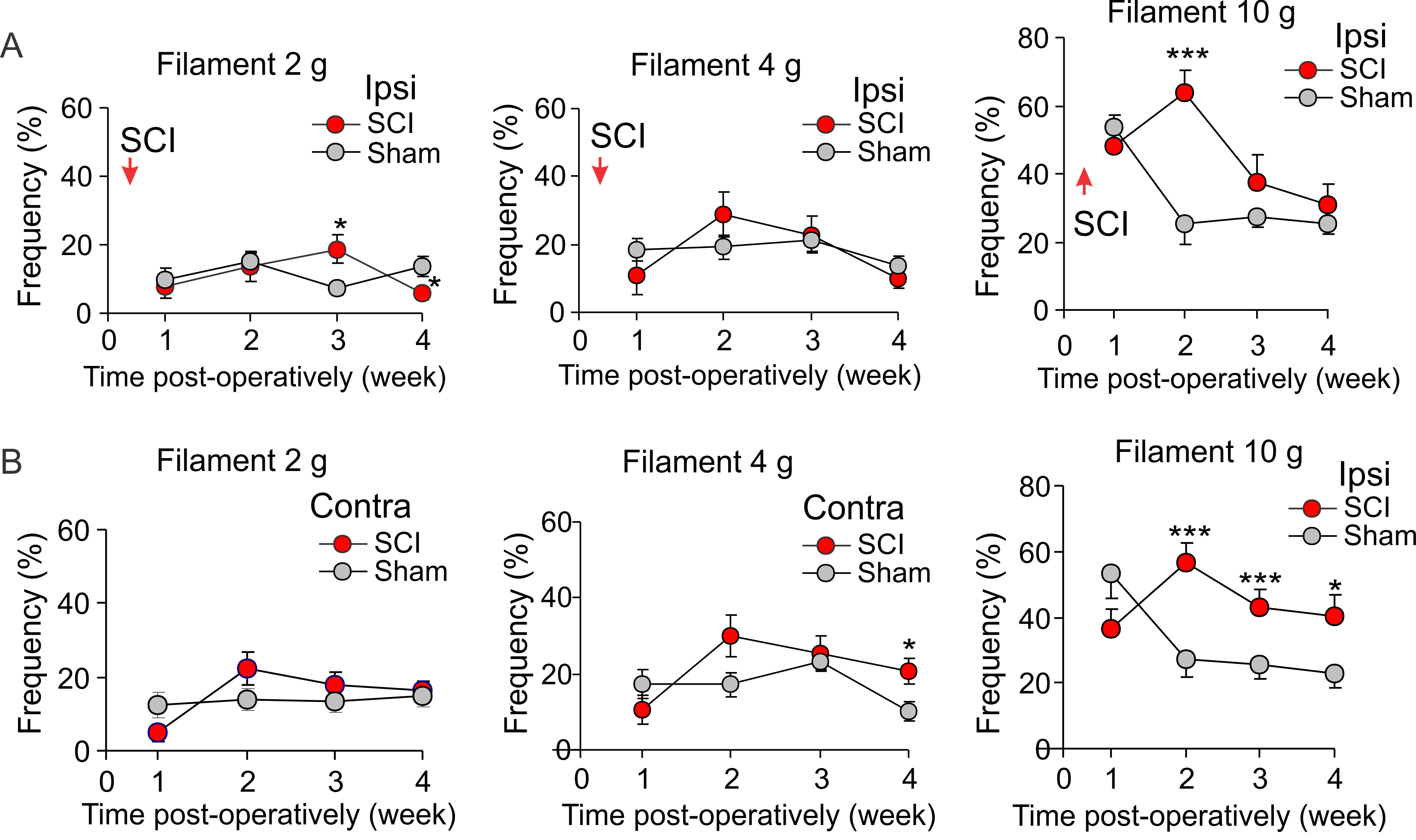


**Figure S2.** The behavioural testing with von Frey monofilaments failed to detect the development of peripheral tactile allodynia in the SCI-injured animals, related to Figure 1.

(A–B) The time course of changes in the paw withdrawal frequency in response to the mechanical stimulation with von Frey monofilaments of different stimulus intensity (subthreshold, the 2-g and 4-g von Frey monofilaments; suprathreshold, the 10-g von Frey monofilaments) on the ipsilateral (A) and the contralateral (B) to hemisection sides in sham-operated group and the SCI-injured animals.

Data are expressed as mean; error bars, SEM. **p* < 0.05, ****p* < 0.01 compared to the corresponding time in sham-operated control.

**
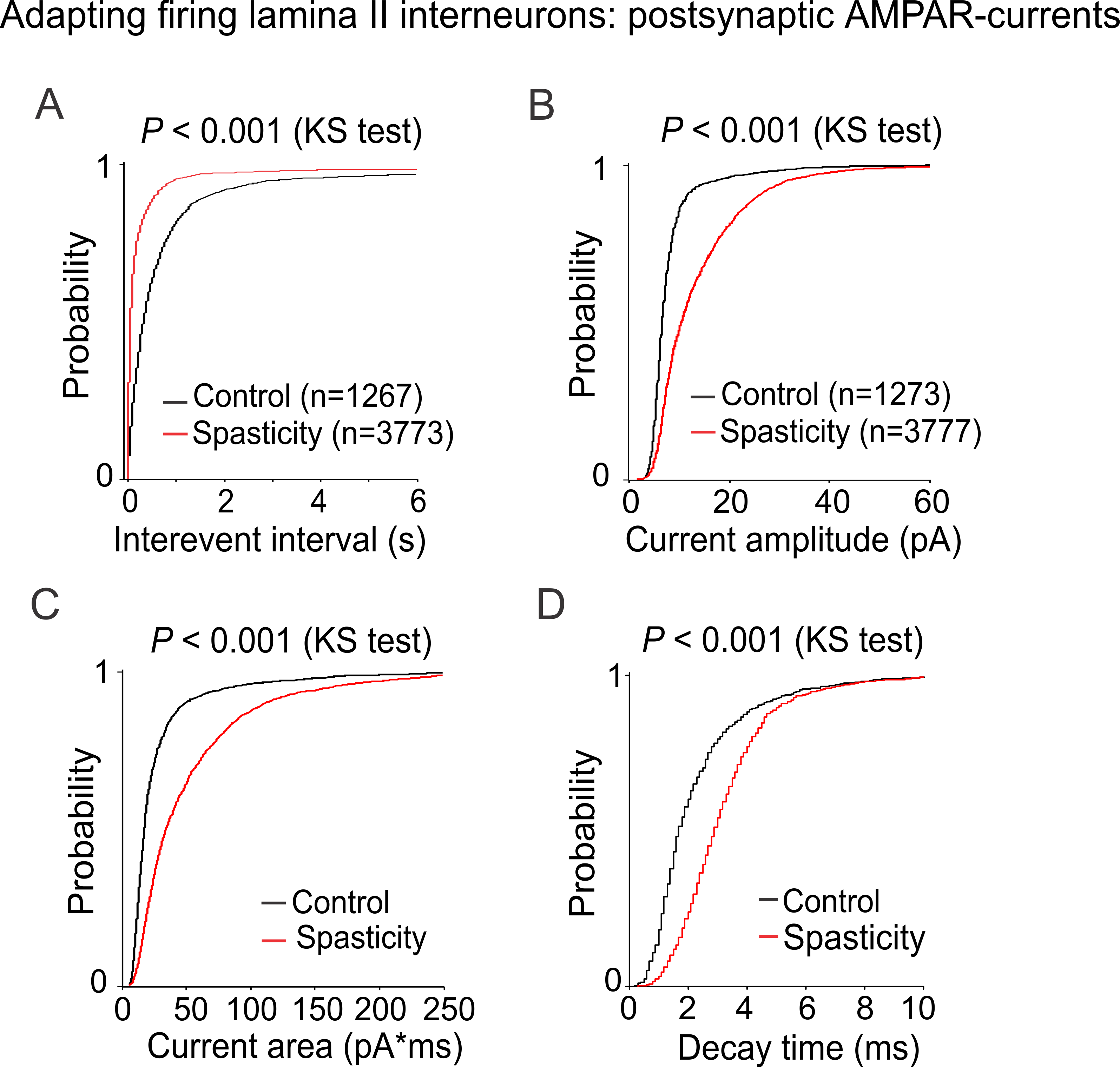
**

**Figure S3.** The upregulated AMPAR-mediated mEPSCs in AFNs post-SCI, related to Figure 3.

Cumulative probability plots for the interevent interval (A), the amplitude (B), the area (C) and the decay kinetics (D) of the AMPAR-mediated mEPSCs in AFNs show differences between conditions.

The Kolmogorov-Smirnov (KS) test significance is indicated.

**
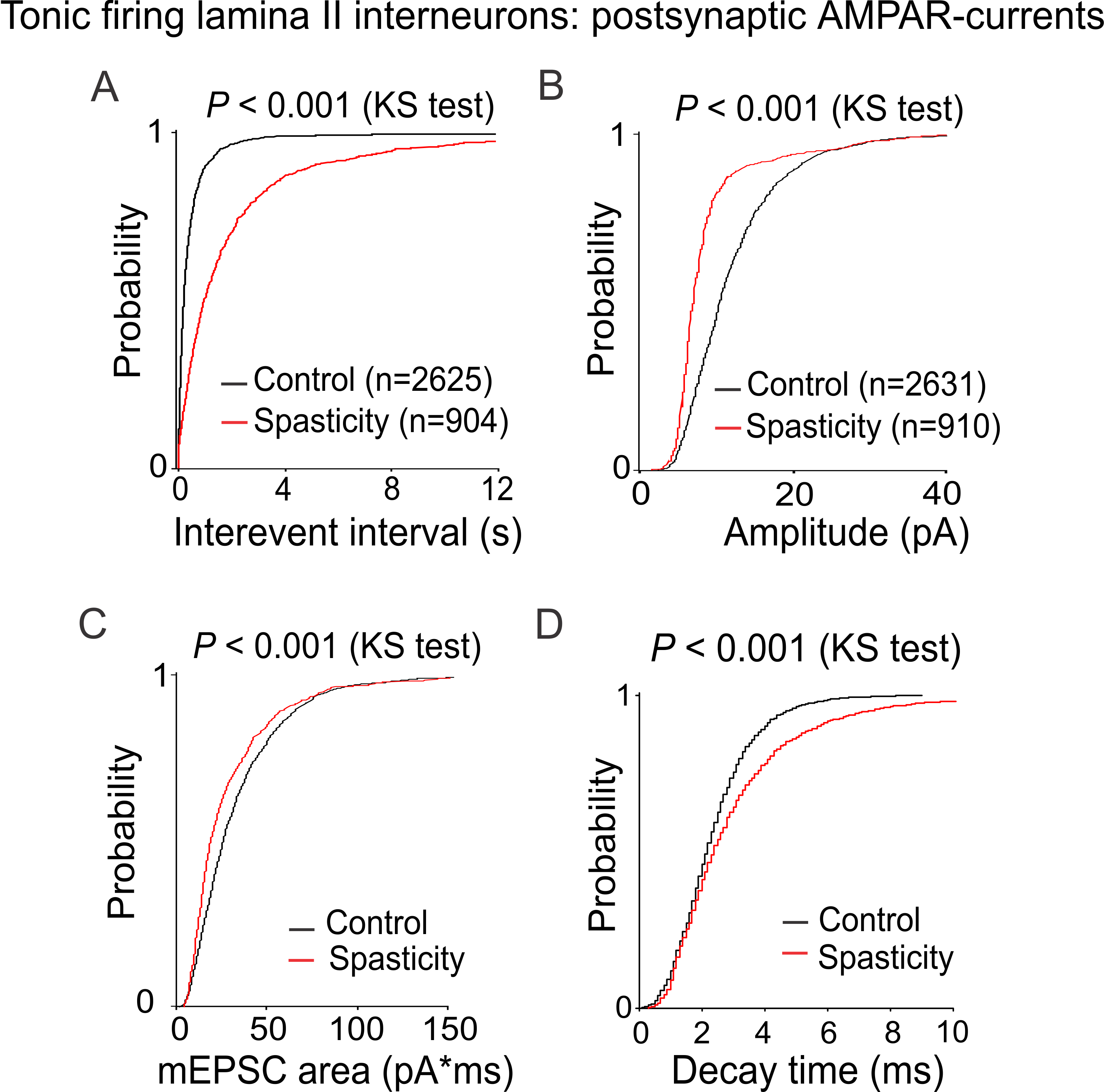
**

**Figure S4.** The downregulated AMPAR-mediated mEPSCs in TFNs post-SCI, related to Figure 4.

Cumulative probability plots for the interevent interval (A), the amplitude (B), the area (C) and the decay kinetics (D) of the AMPAR-mediated mEPSCs in TFNs show the difference between control and spastic conditions, with the changes opposite to those observed in AFNs.

The Kolmogorov-Smirnov (KS) test significance is indicated.

**Extended Experimental Procedures**

**Self-mutilatory behaviour**

A self-mutilatory behaviour (autotomy) has been proposed as a readout of neuropathic pain in animal studies. Therefore, we thoroughly monitored all animals for any signs of autotomy postoperatively, an outcome measure of chronic and intolerable pain post-SCI. Once an animal exhibited attempts to autotomy, progressive for a few days, it has been eliminated for the ethical reasons; these animals were not included into other behavioural assessments.

**The method of von Frey monofilaments**

For assessment of peripheral sensitivity deficit that can develop after SCI, the method of von Frey monofilaments was applied as previously described[^3-5^](#_ENREF_3). Briefly, the animal was placed and allowed to habituate to a Plexiglas chamber on an elevated mesh screen. Von Frey monofilaments of different intensity of stimulus (Bioseb, Italy) were applied to each hind paw. Trial was repeated 10 times for each hind paw with an interval between filament applications for at least 1 minute. The percentage of response (characterized as withdrawing, shaking or licking the hind paw) was calculated for each trial and was defined as the withdrawal frequency.

**Extended references**

1 Abdulla, F. A. & Smith, P. A. Changes in Na(+) channel currents of rat dorsal root ganglion neurons following axotomy and axotomy-induced autotomy. *Journal of neurophysiology* **88**, 2518-2529, doi:10.1152/jn.00913.2001 (2002).

2 Christensen, M. D., Everhart, A. W., Pickelman, J. T. & Hulsebosch, C. E. Mechanical and thermal allodynia in chronic central pain following spinal cord injury. *Pain* **68**, 97-107 (1996).

3 Kopach, O. *et al.* PKCalpha is required for inflammation-induced trafficking of extrasynaptic AMPA receptors in tonically firing lamina II dorsal horn neurons during the maintenance of persistent inflammatory pain. *The journal of pain : official journal of the American Pain Society* **14**, 182-192, doi:10.1016/j.jpain.2012.10.015 (2013).

4 Kopach, O., Viatchenko-Karpinski, V., Belan, P. & Voitenko, N. Development of inflammation-induced hyperalgesia and allodynia is associated with the upregulation of extrasynaptic AMPA receptors in tonically firing lamina II dorsal horn neurons. *Frontiers in physiology* **3**, 391, doi:10.3389/fphys.2012.00391 (2012).

5 Kopach, O., Krotov, V., Goncharenko, J. & Voitenko, N. Inhibition of Spinal Ca(2+)-Permeable AMPA Receptors with Dicationic Compounds Alleviates Persistent Inflammatory Pain without Adverse Effects. *Frontiers in cellular neuroscience* **10**, 50, doi:10.3389/fncel.2016.00050 (2016).
